# Evaluation of Commercially Available Kits for Parallel DNA and microRNA Isolation Suitable for Epigenetic Analyses from Cell-Free Saliva and Salivary Extracellular Vesicles

**DOI:** 10.3390/ijms26136365

**Published:** 2025-07-02

**Authors:** Iqra Yousaf, Ulrike Kegler, Manuela Hofner, Christa Noehammer

**Affiliations:** Center for Health & Bioresources, Molecular Diagnostics, AIT Austrian Institute of Technology GmbH, Giefinggasse 4, 1210 Vienna, Austria; iqrayousaf1991@gmail.com (I.Y.);

**Keywords:** cell-free saliva, extracellular vesicles (EVs), DNA isolation, microRNA isolation, EV isolation, extracellular nucleic acids, disease diagnostics, biomarker, non-invasive

## Abstract

Circulating cell-free nucleic acids (NAs), in particular plasma-derived cell-free DNA, have evolved into promising clinical analytes for prenatal diagnostics, cancer analysis, and cancer surveillance and therapy monitoring. Nevertheless, salivary extracellular and extracellular vesicle (EV)-derived DNA and microRNA have recently gained attention as potential non-invasive biomarkers for a variety of diseases, including cancer, cardiovascular, autoimmune, and infectious diseases. Our goal in this study was therefore to evaluate and optimize commercially available approaches for cell-free nucleic acid isolation, focusing specifically on DNA and miRNA present in cell-free saliva or saliva-derived EVs. Along these lines, we investigated various commercially available kits, which enable parallel isolation of cell-free DNA and RNA in separate fractions from cell-free saliva and salivary EVs, respectively, and compared them to single analyte extraction kits. The efficiency of all tested nucleic acid extraction methods was determined by comparing DNA and RNA fluorescence spectroscopy measurements and quantitative PCR values obtained from a selection of different DNA- and microRNA targets. We found the Norgen Plasma/Serum RNA/DNA Purification Mini kit in combination with the miRCURY exosome isolation kit to work best in our hands and to provide the highest yields of EV-derived nucleic acids. Having tested and identified effective protocols for isolating salivary extracellular nucleic acids, we present with this comparison study, among others, a sound basis for future circulating small nucleic acid and epigenetic biomarker research aiming for early disease diagnosis, prognosis, and prediction from cell-free saliva, representing an easy-to-collect and readily available diagnostic fluid.

## 1. Introduction

Extracellular vesicles (EVs) are a heterogeneous group of membrane-surrounded vesicles that are secreted by cells into the extracellular space and can be found in virtually all human body fluids, such as plasma, serum, urine, cerebrospinal fluid, breast milk, or tears [1]. EVs serve as vehicles for the transfer of molecular cargo, including proteins, nucleic acids, metabolites, and lipids, and thereby significantly contribute to cell-to-cell communication [2,3]. EVs are typically divided into three groups based on their distinctive biogenesis pathways, namely apoptotic bodies, microvesicles, and exosomes [4,5]. Exosomes, nowadays more accurately referred to as small EVs (sEVs), have been successfully isolated from various body fluids such as blood, urine, or saliva. As microRNAs (miRNAs) are abundant in sEVs and play a key role in regulating gene expression by binding to target mRNAs—either inhibiting translation or promoting mRNA degradation—the miRNA repertoire of sEVs has been intensively studied to identify disease-specific biomarkers [6,7]. EVs represent a promising source of biomarkers due to their lipid bilayer, which enables EVs to protect their cargo biomolecules and allows the cargo analytes to persist in the extracellular space without undergoing degradation [1,2]. Another outstanding characteristic highlighting the tremendous potential of EVs to be used for the diagnosis and prognosis of diseases is their high abundance in bodily fluids, especially when compared to circulating tumor cells.

Saliva has been regarded as a reliable and promising biological matrix for early disease diagnosis for some time [8], and the advantages of saliva sampling (non-invasive, repeatedly available, safe, and easy to collect, as no skilled personnel is required) have sparked a lot of interest in the realm of public health—a trend further reinforced by the COVID-19 pandemic. The use of saliva as a biomarker source has clear advantages over other biofluids. Salivary miRNAs are particularly ideal biomarker candidates as they are preserved from enzymatic degradation due to their short length and association with lipoprotein, Ago2 proteins, or EVs [6]. Weber et al. investigated the microRNA spectrum in 12 different body fluids and, among others, state in their publication [9] that the ideal biomarker must be accessible using noninvasive protocols, inexpensive to quantify, specific to the disease of interest, and, in the early disease diagnosis setting, provide a reliable early indication of disease before clinical symptoms appear, a criterion that salivary miRNAs potentially fulfill perfectly. Saliva, as an easy and non-invasive sample matrix to obtain, is not least an attractive source for extracellular vesicles, which are ideal targets of multiomics diagnostic biomarker research, as they most efficiently protect any type of biomolecule engulfed by them and provide information on the status of the cell they derive from [2].

The purpose of the study was the evaluation and optimization of nucleic acid isolation protocols specifically suited for the parallel isolation of small RNA and DNA from cell-free saliva or saliva-derived sEVs. To identify the procedure that is most suitable in terms of effectiveness, time, and costs for future salivary biomarker research, we tested and compared a total of 11 commercial miRNA/DNA extraction kits along with 5 different EV preparation methods and challenged our comparison study by limiting the starting saliva input volume to only 500 μL.

## 2. Results

**Figure 1:**
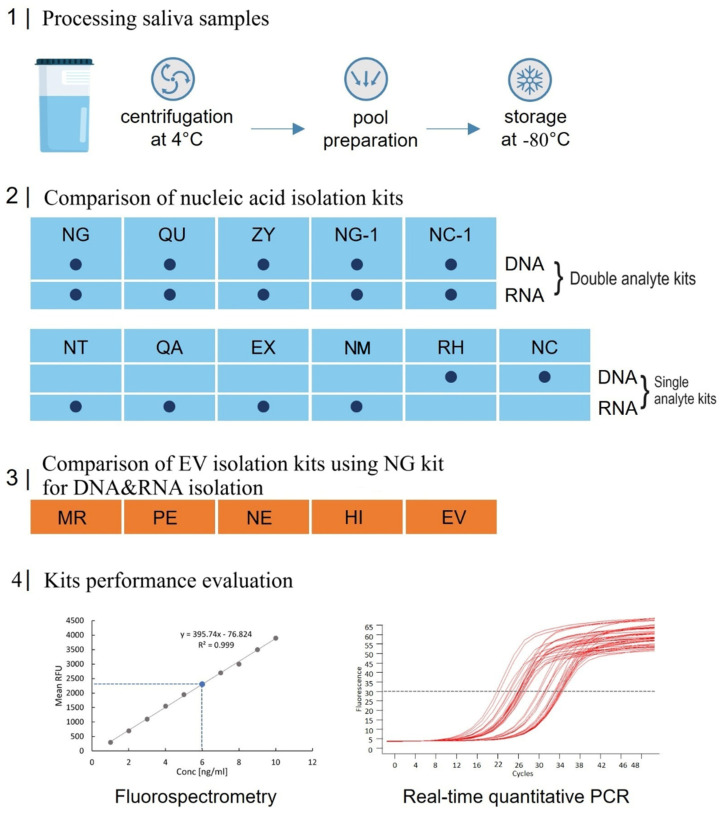
Scheme and flowchart of the study design. Blue rectangles represent the various nucleic acid (NA) isolation kits, and orange rectangles indicate the extracellular vesicle (EV) isolation kits. For abbreviations and detailed descriptions of the kits used, refer to Table 1, Table 2 and Table 3.

An overall scheme of the present study, starting with the collection of saliva samples and concluding with the quantification of isolated DNA and miRNA via quantitative PCR (qPCR), is illustrated in Figure 1. Unstimulated whole saliva was collected from 10 healthy volunteers, 5 females and 5 males after the approval of the local ethical committee. A pool of cell-free saliva was generated from all saliva donors after centrifugation and frozen in 500 uL aliquots at −80 °C for short storage until processing. Nucleic acid isolation (NA) efficiency was tested from 5 commercially available kits for simultaneous isolation of DNA and RNA and compared to the performance of 4 RNA-only—and 2 DNA-only isolation kits. To evaluate and compare a total of 5 different sEV isolation principles in their performance, the Norgen Plasma/Serum RNA/DNA Purification Mini Kit was used for EV NA isolation. After the isolation of cell-free saliva NAs and sEV-derived NAs, nucleic acid concentration was routinely assessed via Fluorescence-based Nanodrop (fluorospectrometry) and qPCR.

### 2.1. DNA Extraction from Cell-Free Saliva

**Figure 2:**
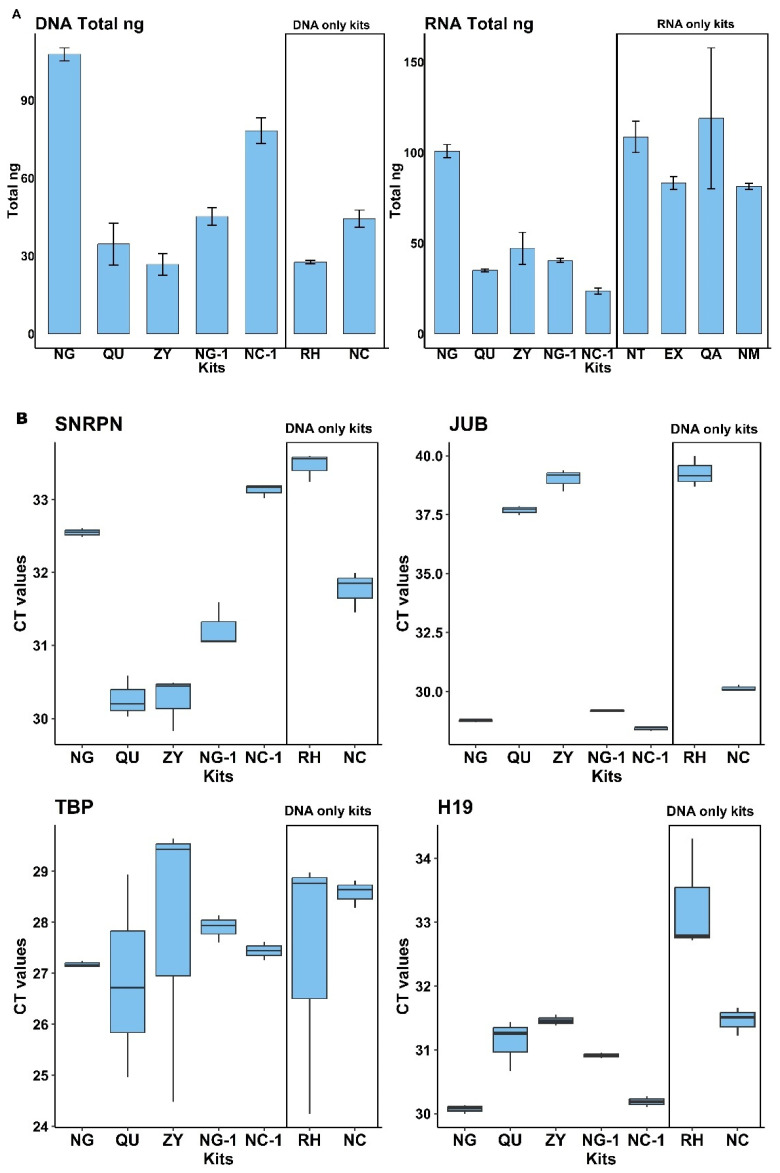
Comparison of commercial kits for DNA extraction: (**A**). Fluorospectrometric quantification of DNA using PicoGreen dye (**left**) and RNA using RiboGreen dye (**right**). Values represent the mean total DNA and RNA concentrations (in ng) ± standard deviation, obtained from 500 µL of cell-free saliva. (**B**). Boxplots showing median Ct values from qPCR targeting four genomic loci (SNRPN, JUB, TBP, and H19). Among the kits designed for simultaneous DNA and RNA isolation, the Norgen (NG) kit yielded the highest DNA amounts, as indicated by the lowest Ct values across most targets.

We evaluated a total of 7 different commercially available isolation kits (see Table 1) and measured the amounts of DNA isolated from cell-free saliva. The starting saliva samples came from a common saliva pool, which was generated by pooling saliva collected from 10 healthy individuals (5 females and 5 males). Before DNA extraction, the saliva samples were subjected to high-speed centrifugation to obtain cell-free saliva (for details, see Section 4). For each kit, we followed the manufacturers’ procedures and recommendations with slight adaptations in the starting volume (500 µL was used for all tested kits) and final elution volume (60 µL for all kits) (for details, see Section 4). All kits were tested in triplicate. Among all extraction kits, the Plasma/Serum RNA/DNA Purification Mini Kit from Norgen (NG) showed the highest total yield of DNA when measured via fluorescence spectroscopy (Figure 2A, left part). We further evaluated and compared the DNA extraction efficiency of the various kits via qPCR for different genomic targets: SNRPN, JUB, TBP, and H19 (Figure 2B). As the Norgen (NG) kit performed best for 3 out of the 4 selected targets in qPCR and offers the unique possibility to isolate simultaneously DNA, RNA, and miRNA in two separate elution fractions from the same cell-free saliva sample, it represents the most promising and valuable option for salivary NA biomarker research and potential clinical diagnostic applications.

**Table 1:** Overview of commercial nucleic acid (NA) isolation kits—both single- and dual-analyte—tested and compared for DNA isolation efficiency from 0.5 mL of cell-free saliva.

Manufacturer	Abbreviation	Kit Name	Type of NA Isolated
Norgen-Biotek Corp., Thorold, ON, Canada	NG	Plasma/Serum RNA/DNA Purification Mini Kit	m/miRNA and DNA
Qiagen., Hilden, Germany	QU	Qiagen AllPrep^®^ DNA/RNA/miRNA Universal	m/miRNA and DNA
Zymo Research Corporation., Tustin, CA, USA	ZY	Zymo ZR-Duet™ DNA/RNA MiniPrep Plus	m/miRNA and DNA
Norgen-Biotek Corp., Thorold, ON, Canada	NG-1	Norgen RNA/DNA Purification Kit	m/miRNA and DNA
Norgen-Biotek Corp, Thorold, ON, Canada	NC-1	Norgen Plasma/Serum Circulating and Exosomal RNA and DNA Purification Kit	m/miRNA and DNA
Roche Diagnostics Deutschland GmbH., Mannheim, Germany	RH	Roche High Pure PCR Template Preparation kit	DNA only
Norgen-Biotek Corp., Thorold, ON, Canada	NC	Norgen Plasma/Serum Cell Free Circulating DNA	DNA only

### 2.2. miRNA Extraction from Cell-Free Saliva

**Figure 3:**
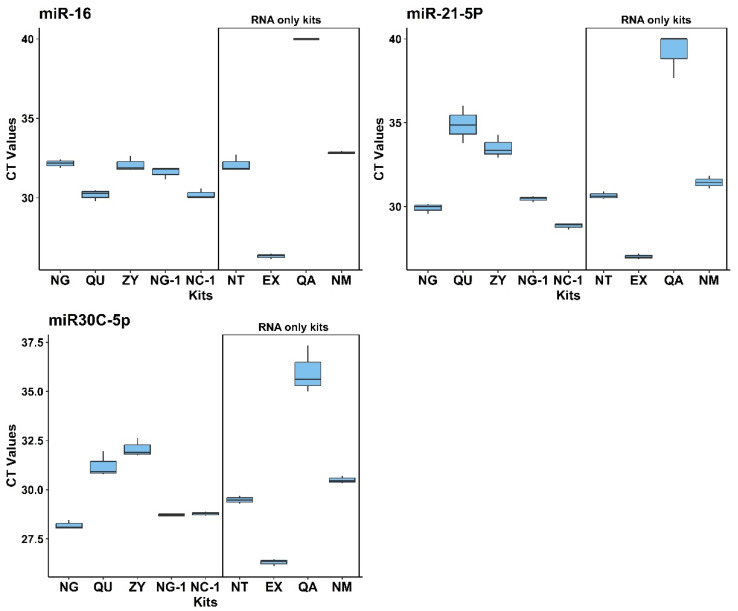
Comparison of miRNA extraction kits—Boxplots of qPCR mean Ct values results. Data are shown for 3 selected miRNA markers (miRNA-16, miRNA-21, and miRNA-30c-5p). Among the double analyte kit NG kits, outperforms the others, as it shows lowest CT values for most miRNA targets.

In addition to DNA isolation efficiency described above, 9 different commercially available NA isolation kits (see Table 2 for an overview) were evaluated for their performance in isolating circulating miRNAs from cell-free saliva. The Norgen Plasma/Serum RNA/DNA Purification Mini Kit (NG) was again one of the most efficient kits in terms of overall RNA yield, whereas the Norgen Plasma/Serum Circulating and Exosomal RNA and DNA Purification Kit (NC-1) resulted in the lowest amount of isolated RNA (Figure 2A right). In addition to fluorescence spectroscopy measurements for RNA yields (as shown in Figure 2A right), miRNA extraction efficiency was evaluated for every single and double analyte kit by multiplex stem-loop qPCR reaction targeting for a set of 3 different miRNA entities: miR-16, miR-21-5p, and miR-30c-5p (Figure 3), which were chosen due to their well-documented abundance and stability in saliva and the fact that stem-loop qPCR assays were already established for them in our group before. Whereas this study focused only on yield and qPCR-based performance metrics, nucleic acid isolation from, e.g., patients’ saliva should always incorporate nucleic acid integrity and fragment size assessments (e.g., via Bioanalyzer, TapeStation, or FragmentAnalyzer) to further validate the suitability of isolated DNA and miRNAs for downstream applications such as next-generation sequencing and epigenetic profiling. As evident from Figure 3, the Exqion miRCURY™ RNA Isolation Kit—Cell & Plant (EX) demonstrated extraordinary performance for all 3 selected miRNA targets. Unfortunately, this kit is no longer available on the market, and, in addition, it supports RNA isolation only. The focus was therefore on the other readily available kits to choose the best-performing one for diagnostic testing purposes. Even though there was considerable variability between the kits across the different miRNA targets, the Norgen Plasma/Serum RNA/DNA Purification Mini Kit (NG) performed again among the top. As this kit resulted in consistently high levels of both miRNAs and DNA, we concluded that it is a highly valuable option for parallel DNA and miRNA isolation from cell-free saliva.

**Table 2:** Overview of (single- and double-analyte) commercial NA kits tested and compared for miRNA isolation from 0.5 mL of cell-free saliva.

Manufacturer	Abbreviation	Kit Name	Type of NA Isolated
Norgen-Biotek Corp., Thorold, ON, Canada	NG	Plasma/Serum RNA/DNA Purification Mini Kit	m/miRNA and DNA
Qiagen, Hilden, Germany	QU	Qiagen AllPrep^®^ DNA/RNA/miRNA Universal	m/miRNA and DNA
Zymo Research Corporation., Tustin, CA, USA	ZY	Zymo ZR-Duet™ DNA/RNA MiniPrep Plus	m/miRNA and DNA
Norgen-Biotek Corp., Thorold, ON, Canada	NG-1	Norgen RNA/DNA Purification Kit	m/miRNA and DNA
Norgen-Biotek Corp., Thorold, ON, Canada	NC-1	Norgen Plasma/Serum Circulating and Exosomal RNA and DNA Purification Kit	m/miRNA and DNA
Norgen-Biotek Corp., Thorold, ON, Canada	NT	Norgen Total RNA Purification Kit	m/miRNA only
Exqion, Vedbæk, Denmark	EX	Exqion miRCURY™ RNA Isolation Kit—Cell & Plant	m/miRNA only
Qiagen, Hilden, Germany	QA	miRNeasy Serum/Plasma Advanced Kit	m/miRNA only
Norgen-Biotek Corp., Thorold, ON, Canada	NM	Plasma/Serum RNA Purification Mini Kit	m/miRNA only

### 2.3. Evaluation of Commercial Kits for Extracellular Vesicle Isolation from Saliva

As extracellular vesicles (EVs) are increasingly being used as a novel reservoir for disease biomarker discovery, we tested 5 different commercially available exosome/EV isolation kits (see Table 3), which covered a range of different isolation principles, such as precipitation, purification via columns, or magnetic bead capture. To be able to compare the EV isolation efficiency of the tested kits, we combined every EV kit with subsequent NA isolation using the NG DNA and RNA isolation kit.

**Figure 4:**
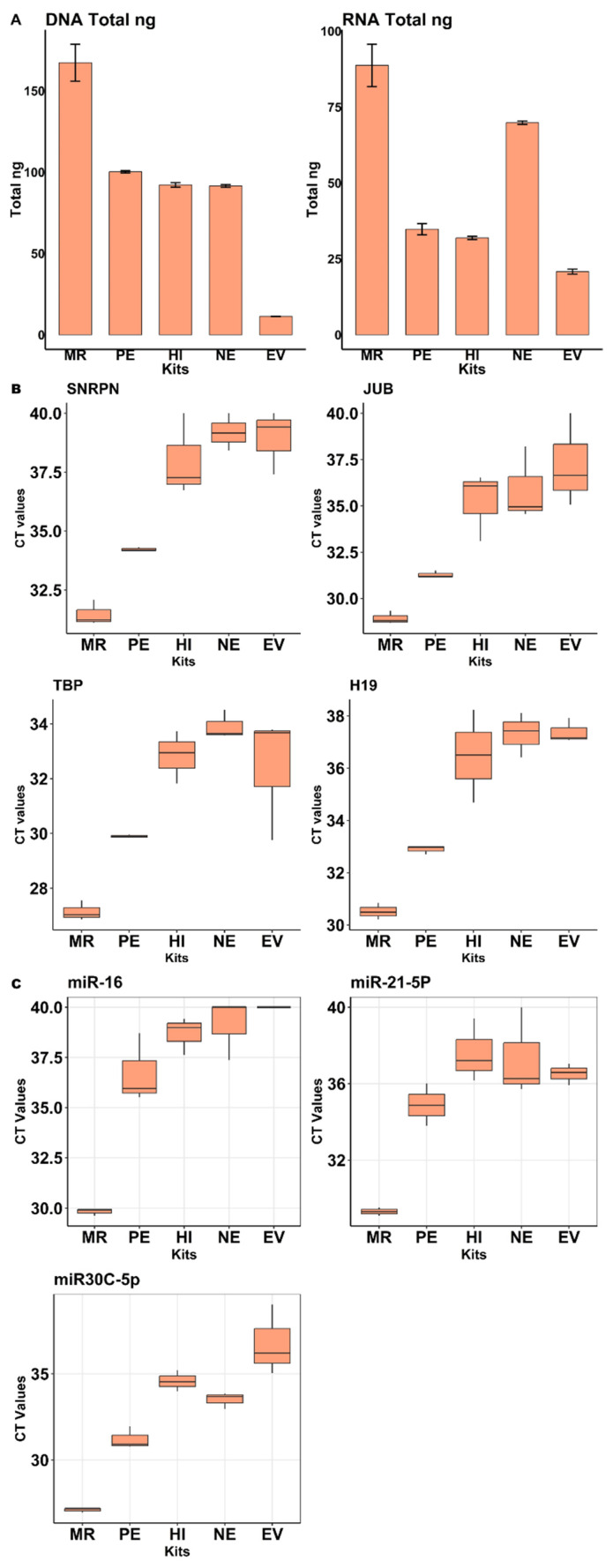
Evaluation of EV isolation kits: (**A**). Fluorospectrometric quantification of DNA (PicoGreen) and RNA (RiboGreen). Values represent the mean total concentrations (ng) of DNA and RNA obtained from salivary EV samples. (**B**). Boxplots showing mean Ct values from qPCR for four DNA markers (SNRPN, JUB, TBP, H19). (**C**). Boxplots showing mean Ct values from qPCR for three miRNA markers (miR-16, miR-21-5p, miR-30c-5p). In conclusion, the miRCURY exosome serum plasma kit in combination with the NG nucleic acid isolation kit resulted in the highest DNA and RNA yields and additionally performed best when it comes to qPCR amplification of selected DNA and miRNA targets as compared to the other EV isolation kits.

According to our quantification results obtained via fluorescence spectroscopy (Figure 4A left), DNA isolation from EVs was most successful using the miRCURY Exosome Serum/Plasma Kit (MR). On the other hand, the EVtrap magnetic capture of extracellular vesicles (EVs) resulted in the lowest DNA yields. By using qPCR for a set of 4 DNA markers (SNRPN, JUB, TBP, and H19), we additionally evaluated and compared the effectiveness of DNA extraction from the various EV isolation kits. The MiRCURY Exosome Serum/Plasma Kit (MR) in combination with the NG NA isolation kit performed best, yielding the lowest Ct values for all 4 gene markers (Figure 4B), which is consistent with the spectroscopy results. When analyzing RNA via fluorospectrometry, again the miRCURY Exosome Serum/Plasma Kit (MR) proved to be the most effective EV isolation method (Figure 4A right), whereas the EVtrap magnetic capture of extracellular vesicles (EVs) was once again the least effective kit for isolation of EVs. By using a multiplex stem-loop qPCR reaction targeting a set of 3 miRNA entities (miR-16, miR-21-5p, and miR-30c-5p), the miRCURY Exosome Serum/Plasma Kit (MR) was demonstrated to perform best for EV RNA isolation, as it again showed the lowest Ct values for the 3 investigated miRNAs (Figure 4C), which were chosen due to their well-documented abundance and stability in saliva and the fact that stem-loop qPCR assays were already established for them in our group before.

**Table 3:** Overview of the investigated, commercially available kits for EV isolation, all tested using a starting input volume of 0.5 mL cell-free saliva.

Manufacturer	Kit Code	Kit Name	Principle
Qiagen, Hilden, Ger-many	MR	miRCURY^®^ Exosome Serum/Plasma Kit	Precipitation
101 Bio., Mountain View, CA, USA	PE	PureExo^®^ Exosome Isolation kit	Precipitation
HansaBioMed Life Sciences., Tallinn, Estonia.	HI	Immunobeads for Overall exosomes capture from biological fluids	Immuno bead-based
Norgen-Biotek Corp., Thorold, ON, Canada	NE	Norgen plasma serum exosomes Purification kit	Column Purification
Tymora Analytical Operations, West Lafayette, IN, USA	EV	EVtrap magnetic capture of extracellular vesicles (EVs)	EV capture via magnetic beads

## 3. Discussion

The isolation of cell-free nucleic acids (NAs) from saliva and salivary extracellular vesicles (EVs) has emerged as a promising strategy for non-invasive biomarker discovery, particularly in the context of early disease diagnosis and monitoring [10]. This study evaluated several commercially available kits for the parallel isolation of DNA and miRNA from cell-free saliva and salivary EVs, with the goal of identifying the most efficient protocols for future genetic, epigenetic, and miRNA/RNA biomarker research. The results demonstrate that the Norgen Plasma/Serum RNA/DNA Purification Mini Kit (NG) outperformed other kits in terms of DNA and miRNA isolation efficiency, particularly when combined with the miRCURY Exosome Serum/Plasma Kit (MR) for EV isolation. These findings have significant implications for the field of non-invasive diagnostics, as they provide a robust foundation for the use of saliva as a diagnostic fluid, which is both easy to collect and rich in biomolecular information [10].

The superior performance of the Norgen NG kit in isolating both DNA and miRNA from cell-free saliva can be attributed to its ability to handle small volumes of starting material while maintaining high yields and purity. This is particularly important in clinical settings where sample volumes may be limited, and efficient use of available material is critical [11]. The kit’s dual isolation capability, which allows for the simultaneous extraction of DNA and RNA, is a significant advantage for comprehensive biomarker studies that require multi-analyte profiling. The high DNA yields observed with the NG kit, as confirmed by both fluorospectrometry and qPCR, suggest that it is well-suited for downstream applications such as next-generation sequencing (NGS) and epigenetic analyses [12]. Furthermore, the kit’s ability to isolate miRNA with high efficiency, as evident from the low Ct values obtained in qPCR, underscores its potential for miRNA-based biomarker discovery. miRNAs are increasingly recognized for their role in regulating gene expression and their association with various diseases, making them ideal candidates for non-invasive diagnostics [9].

The combination of the NG kit with the miRCURY MR kit for EV isolation further enhances its utility in biomarker research. EVs, particularly exosomes, have gained significant attention as reservoirs of disease-specific biomarkers due to their ability to protect their cargo from degradation and their role in intercellular communication [13,14]. The MR kit’s superior performance in isolating EVs, as demonstrated by the high yields of both DNA and RNA, aligns with previous studies that have emphasized the importance of efficient EV isolation methods for biomarker discovery [15,16]. The low Ct values obtained for both DNA and miRNA targets from EV-derived NAs suggest that the MR kit effectively preserves the integrity of the nucleic acids, which is critical for accurate downstream analyses. This is particularly relevant for studies focusing on cancer and other diseases where EV-derived biomarkers are increasingly being explored for their diagnostic and prognostic potential [17,18].

The variability observed in the performance of different kits underscores the importance of selecting appropriate isolation methods based on the specific requirements of the study. For instance, while the Exiqon miRCURY™ RNA Isolation Kit (EX) demonstrated exceptional performance for miRNA isolation, its unavailability on the market limits its practical utility. This highlights the need for continuous evaluation and optimization of commercial kits to ensure that researchers have access to reliable and effective tools for NA isolation. The findings of this study also emphasize the importance of considering the starting sample volume, as the kits were tested with a limited input of 500 µL of saliva. This is particularly relevant for clinical applications where sample availability may be restricted, and efficient use of limited material is crucial.

The use of saliva as a diagnostic fluid offers several advantages over traditional biofluids such as blood or urine, including its non-invasive collection, ease of access, and the ability to collect repeated samples over time. These attributes make saliva an attractive option for large-scale screening programs and longitudinal studies, particularly in the context of early disease detection and monitoring [19]. The successful isolation of high-quality NAs from saliva and salivary EVs, as demonstrated in this study, further supports the potential of saliva as a reliable source of biomarkers for a wide range of diseases, including cancer, cardiovascular diseases, and infectious diseases [20,21]. Moreover, the ability to isolate both DNA and miRNA from the same sample opens up new possibilities for multi-omics approaches, which can provide a more comprehensive understanding of disease mechanisms and improve diagnostic accuracy [19,22].

In comparison to other studies, our research uniquely evaluates the utility of different commercially available kits originally designed for plasma and serum in a novel sample matrix, namely cell-free saliva. While there are no existing studies that directly compare dual-kit approaches for cell-free saliva and salivary extracellular vesicle (EV) isolation, several studies have explored related aspects. For instance, Schweighardt et al. evaluated various commercial kits for dual extraction of DNA and RNA from human body fluids, highlighting the importance of selecting appropriate isolation methods based on specific study requirements [23]. Similarly, Li et al. compared different RNA isolation methods and library construction kits for RNA sequencing in human saliva, emphasizing the challenges posed by the unique characteristics of saliva [24]. Furthermore, Reseco et al. conducted a comprehensive comparison of salivary EV isolation techniques, demonstrating the variability in EV yield and purity across different methods [25]. These studies underline the significance of optimizing isolation protocols for specific sample types and provide valuable insights that complement our findings. Our study contributes to this body of knowledge by specifically focusing on potential biomarker discovery in cell-free saliva and salivary EVs, representing an easy-to-collect and readily available diagnostic fluid.

Looking ahead, future research should focus on several key directions to build on the findings of this study. First, the protocols identified here should be validated in larger clinical cohorts to confirm their reliability and reproducibility across diverse settings and disease indications [26]. This is particularly important for translating these methods into clinical practice, where consistency and accuracy are paramount. Second, the application of these protocols in multi-omics studies should be explored to fully realize the potential of saliva as a diagnostic fluid. Combining DNA, miRNA, and protein analyses from the same sample provides a more holistic view of disease mechanisms and improves diagnostic accuracy. Third, further optimization and standardization of EV isolation methods is needed to enhance the yield and purity of EV-derived NAs, particularly for low-abundance biomarkers. Additionally, preanalytical factors such as saliva collection timing, handling, and storage conditions should be systematically evaluated to support standardization across studies. Finally, automated extraction systems should be explored, which reduce hands-on time and enhance reproducibility. By addressing these research directions, the field can move closer to realizing the full potential of saliva-based diagnostics for early disease detection and personalized medicine.

## 4. Materials and Methods

### 4.1. Saliva Donors and Collection

Saliva collection from healthy donors (working at the Austrian Institute of Technology) was approved by the local ethics committee of the city of Vienna. Whole saliva was collected from 10 healthy individuals (5 males, 5 females) by unstimulated spitting into a sterile 50 mL Falcon tube. The individuals were instructed not to eat, drink, or smoke for at least 1 h prior to saliva sample collection. Ten minutes before starting collection, each donor had to rinse his/her mouth with water without swallowing it. During the spitting process, eating and drinking were not allowed. After a minimum of 15 mL of saliva per donor was collected, samples were centrifuged at 3000× *g* at 4 °C for 20 min, and cell-free supernatant of each sample was carefully transferred and pooled into a sterile glass bottle without interrupting the cell pellet. Aliquots of 1 mL from the pooled cell-free saliva were stored at −80 °C for downstream experiments.

### 4.2. DNA and miRNA Extraction from Cell Free Saliva

DNA and miRNA, respectively, were extracted using a total of 7 and 9 different commercially available isolation kits (see Table 1 for kits specific to DNA isolation and Table 2 for kits specific to RNA). Each kit’s initial input volume was fixed at 0.5 mL cell-free saliva, and the NA elution volume was always set at 60 μL. All samples were processed in accordance with the manufacturer’s specifications, except for the modifications in sample input and elution volume. The purified DNA and RNA/miRNA isolates were stored at −20 °C and −80 °C, respectively.

### 4.3. Extracellular Vesicle Preparation and Isolation

Extracellular vesicles were prepared from cell-free saliva using several techniques and commercial kits (see Table 3). The starting input volume for each kit was set to 0.5 mL of the previously prepared cell-free saliva pool, and all samples were prepared according to the exosome kit manufacturers’ instructions (with slight adjustments due to the given and fixed sample input; see also the methods section). Extracted EVs were resuspended in 300 µL 1x PBS before DNA and RNA/miRNA were subsequently isolated from them using the Plasma/Serum RNA/DNA Purification Mini Kit (NG), which was previously found to work best for parallel DNA and RNA isolations from cell-free saliva. The purified EVs samples were stored at −80 °C for subsequent extraction of DNA and miRNA.

### 4.4. DNA and RNA Concentration Measurements

DNA concentration measurements were done on NanoDrop™ 3300 Fluorospectrometer (Thermo Scientific, Waltham, MA, USA). All the measurements were performed with either Quant-iT™ PicoGreen^®^ dsDNA or Quant-iT™ RiboGreen^®^ RNA Assay Kit (P11496 and R11490, Thermo Fisher Scientific, Waltham, MA, USA) to measure DNA or RNA in solution according to the manufacturer’s protocol. Prior to dealing with actual samples, we used a concentration range of standard curves from 0.025 pg/L to 10,000 pg/L to measure the linear detection range of the standard curve. Lambda DNA was used to create the DNA standard curve, and the RNA standard was created by diluting a ribosomal RNA by 1:2 in PicoGreen/Ribogreen working solution. Depending on the DNA/RNA content in the samples, several dilutions ranging from 1:2 to 1:20 were prepared.

### 4.5. DNA and miRNA Quantification via qPCR

A DNA standard curve was produced from pooled Lambda reference DNA via serial dilution covering a range of 0.01–64 ng/µL. The RNA standard curve was prepared from a human reference RNA (750700, Agilent, Santa Clara, CA, USA) via serial dilution and covered a range of 0.1–100 ng/µL. Both standard curves were diluted in steps of 1:2, indicating that a Cp-value reduction of 1 represents double the amount of nucleic acid detected in the qPCR reaction. The standard row RNA—and saliva RNA samples were reverse transcribed to cDNA using the TaqMan MicroRNA Reverse Transcription Kit (Applied Biosystems™, Thermo Fisher Scientific, Waltham, MA, USA) on a T3000 Thermocycler (Biometra GmbH, Göttingen, Germany) before being applied to qPCR. All qPCRs were performed on 384-well plates using a LightCycler^®^ 480 (Roche, Basel, Switzerland). The accuracy of Ct-values was checked via melting curve analysis of each resulting PCR product. Calibration curves derived from the standard curve values were used for the calculation of absolute DNA and miRNA amounts. Detailed PCR master mix recipe, primer sequences, fragment length, and PCR conditions are shown in Appendix A (Table A1, Table A2, Table A3, Table A4, Table A5 and Table A6).

### 4.6. Data Analysis

Statistical analysis was performed using One-way and two-way ANOVA tests to investigate differences in the amount and efficiency of extracted DNA and RNA between the different extraction kits. Significant differences were further evaluated using the Wilcoxon Signed Rank Test. Using the Wilcoxon Signed Rank Test, all combinations were evaluated [27]. When a significant difference between the different extraction kits was identified, the glht function from the multcomp package was employed to perform Tukey’s post-hoc pairwise comparisons [28]. The *p*-values less than 0.01 were considered as significant. All the statistical analyses were performed using R software version 4.2.2 [29], version 3.6.1. Results are shown in Appendix B, Appendix C and Appendix D (Table A7, Table A8 and Table A9).

## 5. Conclusions

This study systematically compared commercial kits for isolating DNA and miRNA from cell-free saliva and salivary EVs. The Norgen Plasma/Serum RNA/DNA Purification Mini Kit, especially in combination with the miRCURY exosome isolation kit, showed the highest efficiency. Our findings highlight the suitability of these protocols for small nucleic acid biomarker research. Importantly, this work demonstrates the successful adaptation of plasma-based kits to saliva—a non-invasive, easy-to-collect diagnostic fluid. To our knowledge, this is the first comprehensive evaluation of dual DNA/miRNA extraction and EV isolation methods in saliva. These optimized protocols provide a strong foundation for future studies aiming at early disease detection and epigenetic analysis using salivary biomarkers.

## Data Availability

Additional data can be requested from the corresponding author of this study.

## Appendix A

Overview of Primer sequences, fragment length and PCR conditions and master mix recipe for PCR reaction

**Table A1:** Primer sequences DNA targets.

Primer Name	Forward Primer Reverse Primer	Product Length	Cycles
H19	TGG GCC GCA GTG CCT CGT GGG GGG CGA AGC GGC CAC GGG AG	101	45
SNRPN	GTC CCC CAT CCG CCC CCA ACT G CCC ACT GCG GTT ACC CCG CAT GCT C	105	45
JUB	GGC ATT GCT CTG CCC ATA GAT GCC TTT G GGA ATC CCT GGT TTT GAC CTG GGG GA	101	45
TBP	GGC CCG CGG CTC TGT GCG GTG TCG GAT CCG CAG GCG CAG	105	45

**Table A2:** qPCR-Reaction Master mix for DNA.

PCR-Reaction	1 Reaction [µL]		
AD	5.716		
10x Taq-buffer with MgCl (Qiagen)	1.2		
dNTP (2 mM)	0.96		
Eva Green (20x) (Biotium, Fremont, CA, USA)	0.168		
Primer fw (10 µM)	0.24		
Primer rev (10 µM)	0.24		
HotStar Taq Polymerase (Qiagen)	0.072		
Sample	2		
∑ **qPCR-Program**	12 **Temperature**	**Time**	
Activation		5 min	
Denaturation	95 °C	40 s	
Annealing	65 °C	40 s	45x
Elongation	72 °C	1 min 20 s	
Final elongation	72 °C	7 min	

**Table A3:** Primer sequences for miRNA targets.

miRNA	Forward Primer Universal Reverse Primer
miR-16	CGC GCT AGC AGC ACG TAA AT GTG CAG GGT CCG AGG T
miR-21	GCC CGC TAG CTT ATC AGA CTG ATG GTG CAG GGT CCG AGG T
miR-30c-5p	AGC CGC CTG TAA ACA TCC TAC ACT GTG CAG GGT CCG AGG T

**Table A4:** RT-loop sequences.

miR-16	GTCGTATCCAGTGCAGGGTCCGAGGTATTCGCACTGGATACGACCGCCAA
miR-21	GTCGTATCCAGTGCAGGGTCCGAGGTATTCGCACTGGATACGACTCAACA
miR-30c-5p	GTCGTATCCAGTGCAGGGTCCGAGGTATTCGCACTGGATACGACGCTGAG

**Table A5:** RT-Reaction cDNA Master mix.

Master Mix	1 Reaction [µL]		
AD	5.66		
10x buffer	1.5		
100 mM dNTP	0.15		
RNAse Inhibitor (20 U/µL)	0.19		
Mutiscripte RT	1		
RT-loop Primer (20 nM)	1.5		
Master mix	10		
Sample (RNA)	5		
total ∑	15		
**RT-Program**	**Temperature**	**Time**	
Activation	16 °C	30 min	
Denaturation	20 °C	30 s	
Annealing	42 °C	30 s	60x
Elongation	50 °C	1 s	
Final elongation	85 °C	5 min	
Hold	4 °C	hold	

15 µL of cDNA was then diluted by addition of 30 µL of water.

**Table A6:** qPCR-Reaction Master mix for miRNA.

PCR-Reaction	1 Reaction [µL]		
AD	5.716		
10 x Taq-buffer with MgCl (Qiagen)	1.2		
dNTP (2 mM)	0.96		
Eva Green (20x) (Biotium)	0.168		
Primer uni Rev (100 µM)	0.084		
HotStar Taq Polymerase (Qiagen)	0.072		
Primer fw(10 µM)	1.8		
Sample	2		
∑ **qPCR-Program**	12 **Temperature**	**Time**	
Activation		05 min	
Denaturation	95 °C	40 s	
Annealing	60 °C	40 s	45x
Elongation	72 °C	1 min 20 s	
Final elongation	72 °C	7 min	

## Appendix B

**Table A7:** Comparison of cell free salivary DNA isolation performance across all tested extraction kits. Significant *p* values are shown in bold.

	Nanodrop	Cell Free Saliva DNA Gene Markers			
	Picogreen	SNRPN	JUB	TBP	H19
QU x NG	**<0.001**	**<0.001**	**<0.001**	0.999	0.057
ZY x NG	**<0.001**	**<0.001**	**<0.001**	0.999	**0.008**
NG-1 x NG	**<0.001**	**<0.001**	0.675	0.998	0.178
NC-1 x NG	**<0.001**	0.141	0.841	0.999	1.000
RH x NG	**<0.001**	**0.008**	**<0.001**	0.999	**<0.001**
NC x NG	**<0.001**	**0.024**	**0.002**	0.940	**0.008**
ZY x QU	0.356	0.990	**0.003**	0.990	0.924
NG-1 x QU	0.111	**0.005**	**<0.001**	0.987	0.993
NC-1 x QU	**<0.001**	**<0.001**	**<0.001**	0.999	0.108
RH x QU	0.481	**<0.001**	**<0.001**	0.999	**<0.001**
NC x QU	0.166	**<0.001**	**<0.001**	0.866	0.924
NG-1 x ZY	**0.002**	**0.005**	**<0.001**	0.999	0.599
NC-1 x ZY	**<0.001**	**<0.001**	**<0.001**	0.999	**0.016**
RH x ZY	1.000	**<0.001**	0.949	0.999	**<0.001**
NC x ZY	**0.0034**	**<0.001**	**<0.001**	0.998	0.999
NC-1 x NG-1	**<0.001**	**<0.001**	0.118	0.999	0.305
RH x NG-1	**0.003**	**<0.001**	**<0.001**	0.999	**<0.001**
NC x NG-1	0.999	0.203	**0.035**	0.998	0.599
RH x NC-1	**<0.001**	0.666	**<0.001**	0.999	**<0.001**
RH x NG-1	**<0.001**	**<0.001**	**<0.001**	0.977	**0.016**
NC x NG-1	**0.005**	**<0.001**	**<0.001**	0.965	**<0.001**
RH x NC-1	**<0.001**	**<0.001**	**<0.001**	0.999	0.057
NC x NC-1	**<0.001**	**<0.001**	**<0.001**	0.999	**0.008**
NC x RH	**<0.001**	**<0.001**	0.675	0.998	0.178

## Appendix C

**Table A8:** Comparison of cell free salivary miRNA isolation performance across all tested extraction kits. Significant p values are shown in bold.

	Nanodrop	Cell Free Saliva miRNA Gene Markers		
	Riobgreen	miR16	miR21-5p	miR30c-5p
QU x NG	**<0.01**	**<0.001**	**<0.001**	**<0.001**
ZY x NG	**<0.01**	0.999	**<0.001**	**<0.001**
NG-1 x NG	**<0.01**	0.559	0.979	0.928
NC-1 x NG	**<0.01**	**<0.001**	0.565	0.875
NT x NG	0.998	0.999	0.888	0.106
EX x NG	0.806	**<0.001**	**0.001**	**0.005**
QA x NG	0.788	**<0.001**	**<0.001**	**<0.001**
NM x NG	0.720	0.309	0.169	**<0.001**
ZY x QU	0.969	**<0.001**	0.269	0.479
NG-1 x QU	0.999	**0.00162**	**<0.001**	**<0.001**
NC-1 x QU	0.979	0.999	**<0.001**	**<0.001**
NT x QU	**<0.01**	**<0.001**	**<0.001**	**0.01249**
EX x QU	**0.0104**	**<0.001**	**<0.001**	**<0.001**
QA x QU	**<0.01**	**<0.001**	**<0.001**	**<0.001**
NM x QU	**0.0145**	**<0.001**	**<0.001**	0.724
NG-1 x ZY	0.999	0.690	**<0.001**	**<0.001**
NC-1 x ZY	0.504	**<0.001**	**<0.001**	**<0.001**
NT x ZY	**<0.01**	0.999	**0.00137**	**<0.001**
EX x ZY	0.089	**<0.001**	**<0.001**	**<0.001**
QA x ZY	**<0.01**	**<0.001**	**<0.001**	**<0.001**
NM x ZY	0.119	0.220	**0.025**	**0.024**
NC-1 x NG-1	0.843	**0.002**	0.126	0.999
NT x NG-1	**<0.01**	0.676	0.999	0.657
EX x NG-1	**0.0279**	**<0.001**	**<0.001**	**<0.001**
QA x NG-1	**<0.01**	**<0.001**	**<0.001**	**<0.001**
NM x NG-1	**0.0381**	**0.007**	0.665	**0.010**
NT x NC-1	**<0.01**	**<0.001**	0.063	0.7461
EX x NC-1	**<0.01**	**<0.001**	0.060	**<0.001**
QA x NC-1	**<0.01**	**<0.001**	**<0.001**	**<0.001**
NM x NC-1	**<0.01**	**<0.001**	**0.00345**	**0.014**
EX x NT	0.404	**<0.001**	**<0.001**	**<0.001**
QA x NT	0.989	**<0.001**	**<0.001**	**<0.001**
NM x NT	0.324	0.229	0.861	0.310
QA x EX	0.092	**<0.001**	**<0.001**	**<0.001**
NM x EX	1	**<0.001**	**<0.001**	**<0.001**
NM x QA	0.068	**<0.001**	**<0.001**	**<0.001**

## Appendix D

**Table A9:** Comparison of salivary EVs isolation performance across all tested EV kits. Significant *p* values are shown in bold.

	Nanodrop	DNA Gene Marker				Nanodrop	miRNA Gene Marker		
	Picogreen	SNRPN	JUB	TBP	H19	Ribogreen	miR16	miR21-5p	miR30c-5p
HI x EV	**<0.001**	0.820	0.604	0.990	0.747	**0.013**	0.820	0.604	0.990
MR x EV	**<0.001**	**<0.001**	**<0.001**	**0.001**	**<0.001**	**<0.001**	**<0.001**	**<0.001**	**0.001**
NE x EV	**<0.001**	0.998	0.860	0.528	1.000	**<0.001**	0.998	0.860	0.528
PE x EV	**<0.001**	**0.002**	0.010	0.128	**<0.001**	**0.002**	**0.002**	0.010	0.128
MR x HI	**<0.001**	**0.000**	0.007	**<0.001**	**<0.001**	**<0.001**	**0.000**	0.007	**<0.001**
NE x HI	1.000	0.669	0.987	0.779	0.793	**<0.001**	0.669	0.987	0.779
PE x HI	0.367	**0.011**	0.093	0.064	**0.005**	0.815	**0.011**	0.093	0.064
NE x MR	**<0.001**	**<0.001**	0.003	**<0.001**	**<0.001**	**0.000**	**<0.001**	0.003	**<0.001**
PE x MR	**<0.001**	0.065	0.463	0.087	0.060	**<1 × 10^−4^**	0.065	0.463	0.087
PE x NE	0.306	**0.001**	0.044	**0.011**	**0.002**	**<1 × 10^−4^**	**0.001**	0.044	**0.011**
